# C.292G>A, a novel glycine receptor alpha 1 subunit gene *(GLRA1)* mutation found in a Chinese patient with hyperekplexia: A case report

**DOI:** 10.1097/MD.0000000000019968

**Published:** 2020-04-24

**Authors:** Yan Zhang, Ling-Ling Wu, Xiao-Lan Zheng, Cai-Mei Lin

**Affiliations:** Xiamen Children's Hospital, Xiamen, China.

**Keywords:** clonazepam, glycine receptor alpha 1 subunit gene, hyperekplexia, startle

## Abstract

**Introduction:**

Hyperekplexia is a rare hereditary neurological disorder; only 5 glycine receptor alpha 1 subunit gene (*GLRA1*) mutations have been reported in 5 Chinese patients. We report a Chinese infant with hyperekplexia and a novel mutation at c.292G > A.

**Patient concerns:**

A Chinese infant with hyperekplexia and a novel mutation at c.292G > A.

**Diagnosis:**

All exons of *GLRA1* were sequenced in her parents and her, which revealed a mutation at c.1030C > T and another novel mutation at c.292G > A. Her diagnosis was confirmed as hereditary hyperekplexia with *GlRA1* hybrid gene mutations based on the sequencing results.

**Interventions:**

She was treated with clonazepam.

**Outcomes:**

Her muscle hypertonia recovered rapidly and the excessive startle reflex to unexpected stimuli was significantly reduced.

**Conclusion:**

Genetic DNA sequencing is a crucial method for diagnosing hyperekplexia-related gene mutation.

## 1 Introduction

Hyperekplexia, or startle disease, is a rare hereditary neurological disorder characterized by generalized stiffness, excessive startle reflex to unexpected stimuli, and a short period of generalized stiffness following the startle response.^[1]^ It was first reported as “drop seizures” by Kirstein and Silfverskiold in 1958.^[2]^ In 1962, an uncertain hereditary disease that initially manifested with hypertonia was described by Kok and Bruyn. The first genetic analysis of hyperekplexia was carried out in a large Dutch family in 1995 and revealed a mutation within the glycine receptor alpha 1 (*GLRA1*) gene.^[3]^ No epidemiological data for hyperekplexia have been published to date, but more than 200 confirmed cases have been reported globally. Increasing numbers of cases of hyperekplexia have been reported with the increased use of genetic sequencing.^[3–12]^ Only 5 *GLRA1* mutations have been reported in 5 Chinese patients, including 2 cases in Hong Kong,^[13,14]^ 2 cases in 1 family in Taiwan,^[15]^ and 1 case in Beijing.^[9]^ The pathogenesis of hyperekplexia is involved in glycinergic neurotransmission disorders and is associated with *GLRA1*, *GLRB*, *GPHN*, *ARHGEF9*, and *SLC6A5* gene mutations.^[7]^ Infants with hyperekplexia present marked improvement of the startle response and muscle hypertonia upon treatment with clonazepam, which is a strong clinical feature of *GLRA1*-mediated hyperekplexia.

Here, we describe the case of a 4-month-old female with muscle hypertonia. She was initially misdiagnosed with epilepsy due to the excessive startle reflex, but her diagnosis was later confirmed to be hyperekplexia with 2 mutations of the *GLRA1* gene, 1 of them being novel. This study was approved by the ethics committee of the Xiamen Children's Hospital, and written consent for its publication was provided by the patient's parents.

## 2 Case report

A 4-month-old female Chinese infant was suspected of having convulsive seizures due to an excessive startle reflex on the first day of life and admitted to a local hospital. She was the first child of a non-consanguineous family with no family history of neurological disease. She was delivered vaginally at term with a birth weight of 3450 g and no remarkable antenatal abnormalities or birth history. The Apgar scores for the infant were 10, 10, and 10 at 1, 5, and 10 minutes after birth, respectively. After a course of antibiotics, gastric lavage, and other supportive treatments, she continued to be hypertonic and was discharged home. On the 11th day of her life, the symptoms occurred again at a local hospital. Her convulsive seizures were characterized by cyanosis of the lips, salivation, and hypertonicity of the limbs and lasted about 2 minutes before spontaneous resolution. Because no improvement occurred after treatment with phenobarbital for about 4 months, she was sent to our hospital, where we found hypertonicity in response to sudden auditory stimulus. The patient's clinical diagnosis was subsequently revised to hyperekplexia.

The findings on cranial magnetic resonance imaging, full blood count evaluation, C-reactive protein measurement, as well as blood gas and electrolyte analyses were normal. Pulmonary computed tomography suggested left inferior lobe pneumonia. Electroencephalography showed abnormalities with an α-like rhythm in the midline and bilateral frontal areas during both sleep and wakefulness. Several paroxysmal abnormal movements were detected with enhancement of the aforementioned α-like rhythm. In Xiamen Childrenʼs Hospital, physical examination showed a positive result on the nose-tapping test (head-retraction reflex). To exclude epilepsy, video electroencephalography was performed. During the test, some stimuli were presented, but no abnormal waves were induced except for some myoelectric artefacts.

**Figure 1:**
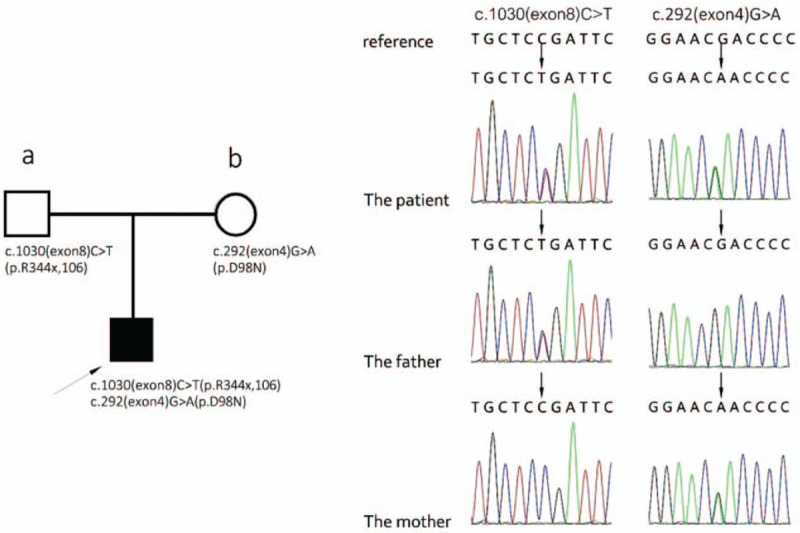
c.1030 (exon8)C > T mutation of *GLRA1* gene and c.292 (exon 4)G > A mutation of *GLRAA1* gene. The whole exon of the *GLRA1* gene was sequenced by Tiro Whole Exome Sequencing in a sample from the patient with hyperekplexia, her father (a), and her mother (b). The c.1030 (exon8)C > T mutation was found in the patient and her father (b), and the c.292(exon 4)G > A mutation was found in the patient and her mother (b).

For genetic analysis, peripheral blood samples were collected from the patient and her parents. Genomic DNA was extracted for Tiro Whole Exome Sequencing. The possible effects of the mutations on GLRA1 protein function were analyzed using SIFT, PROVEAN, MutationTaster, Mendelian Clinically Applicable Pathogenicity, REVEL, GERP, and phastCons20way. Preliminary genetic tests revealed complex heterozygosity of the *GlRA1* gene. One mutation was at c.1030C > T in exon 8 p.R344X,106 (p.Arg344Stop.106), and another was a novel mutation at c.292G > A in exon 4 p.D98N(pAs98Asn) (Fig. 1). The c.1030C > T mutation was also detected in her mother's sample, and the c.292G > A was found in her father's sample (Fig. 1). Therefore, her diagnosis was finally confirmed as hereditary hyperekplexia with *GlRA1* hybrid gene mutations based on the sequencing results.

The patient was treated with clonazepam (0.044 mg/kg/d) three times a day after diagnosis with hyperekplexia. Her muscle hypertonia recovered rapidly, and the excessive startle reflex to unexpected stimuli disappeared by the second day of administration of the medicine. The dose of clonazepam was continually applied until the follow-up 2 months later; at that time, her muscle hypertonia was nearly normal and the startle responses were almost resolved.

## 3 Discussion

The typical clinical manifestations of hyperekplexia are generalized stiffness, excessive startle reflex to unexpected stimuli, and a short period of generalized stiffness following the startle response. Some older children may fall after being frightened. This disease can cause serious injuries due to frequent falls and even can cause infantile death via induced apnea. The patient in the present case had shown obvious muscle hypertonia, excessive startle response to sudden expected auditory, and tactile stimuli and post-startle stiffness since birth.

Hyperekplexia can be hereditary or can occur sporadically. Five genes have been reported to be associated with this condition, namely *GLRA1, SLC6A5, GLRB, GPHN,* and *ARHGEF9*.^[16,17]^ Among them, the *GLRA1* gene accounts for 80% of the hereditary cases. Eight mutations have been detected with *GLRA1* genes, and 20% of the patients with a *GLRA1* mutation had no family history of hyperekplexia. Mutation of *SLC6A5*, which encodes the presynaptic glycine transporter 2, has been found to be the second major cause of hyperekplexia.^[17]^ In the present case, both the patient's father and mother were *GLRA1* mutation gene carriers. Genetic sequencing of the *GLRA1* gene showed 2 mutations [c.1030C > T in exon 8 p.R344X,106 (p.Arg344Stop.106) and c.292G > A in exon 4 p.D98N (pAs98Asn)] in the patient, 1 of which (c.292G > A) is novel. Notably, her healthy mother carried the first variant, whereas her healthy father carried the second variant. C.1030 (exon)C > T has been predicated to be a pathogenic mutation in hyperekplexia,^[2]^ with a change to arginine (AGA) at position 344 of the *GLAR1* gene. This mutation is listed in the Exome Aggregation Consortium (ExAC) depository as MAF = 0.0005 (http://exac.broadinstitute.org). C.292 G > A is a novel mutation of the *GLRA1* gene. Base substitution at the DNA level results in the conversion of 98 amino acids from asparagic acid to asparagine. C.292 G > A is listed in the dbSNP Short Genetic Variations database as rs760390019,0.000016 but not in the ExAC depository. Because both mutations were found in her parents, we therefore conclude that her variant is hereditary.

The nose-tapping test is a specific clinical test for hyperekplexia. The present case did show a positive response on the test. However, in some infants with hyperekplexia, nose-tapping may result in exaggerated startle and stiffening episodes without habituation.^[18]^ Hyperekplexia is easily misdiagnosed as epilepsy because of the manifestations of convulsion and muscle twitching. Indeed, our patient had been diagnosed with seizures preliminarily. The major differential diagnostic disorders include jitteriness, myoclonic seizures, neonatal tetanus, startle epilepsy, and neonatal abstinence syndrome. In terms of treatment, the specific medicine for hyperekplexia is clonazepam, and clonazepam was verified to be effective in the present case. Other drugs, including carbamazepine, phenytoin, diazepam, valproate, 5-hydroxytrytophan, piracetam, and phenobarbital, have been reported to have variable results.^[13]^ Potential life-threatening complications include severe tonic spasms and apnea, which could be treated by the Vigevano maneuver.^[18]^ Therefore, hyperekplexia is potentially treatable. In order to prevent injury and improve the quality of the patient's life, it should be treated as early as possible, because early diagnosis and treatment are important for preventing life-threatening apnea.^[5]^ Prompt genetic analysis of an amniotic fluid sample may be helpful for early definitive diagnosis of hyperekplexia, which will provide enough time for subsequent preconception counseling and safer care.^[9]^ In conclusion, genetic DNA sequencing is a crucial method for diagnosing hyperekplexia and determining the exact gene mutation(s) responsible. This approach could be used to screen for hyperekplexia-related gene mutations in amniotic fluid in cases in which the parents of a fetus have a hyperekplexia-related gene mutation. However, the case was not followed-up because disconnection.

## Acknowledgments

The authors would like to thank the patient and her family for allowing us to use the medical documentation and information that led to the present article.

## Author contributions

**Conceptualization:** Yan Zhang

**Data curation:** Yan Zhang, Ling Ling Wu, Xiao Lan Zheng

**Formal analysis:** Cai-Mei Lin

**Supervision:** Ling Ling Wu

**Validation:** Cai-Mei Lin

**Writing – original draft:** Yan Zhang.

**Writing – review & editing:** Yan Zhang, Ling Ling Wu, Xiao Lan Zheng, Cai-Mei Lin
